# The Effective Separation of Gallium, Vanadium, and Aluminum from a Simulated Bayer Solution by Resin Exchange

**DOI:** 10.3390/ma17164109

**Published:** 2024-08-20

**Authors:** Zhifeng Qin, Xi Jin, Zhen Yang, Yuntao Xin, Weizao Liu

**Affiliations:** 1 State Key Laboratory of Vanadium and Titanium Resources Comprehensive Utilization, Pangang Group Researh Institute Co., Ltd., Panzhihua 617000, China; qzfscu@126.com (Z.Q.); yangzhenyzr@163.com (Z.Y.); 2 Department of Advanced Functional Materials, Chengdu Institute of Advanced Metallic Material Technology and Industry Co., Ltd., Chengdu 610399, China; 3 College of Materials Science and Engineering, Chongqing University, Chongqing 400044, China; jinx@stu.cqu.edu.cn; 4 College of Chemistry and Chemical Engineering, Central South University, No. 932 Lushan South Road, Changsha 410083, China; xinyuntao0707@163.com

**Keywords:** gallium, vanadium, separation, adsorption, ion exchange

## Abstract

The effective recovery of gallium from wastewater discharge in the Bayer process is promising for the long-term development of gallium resources. The adsorption and desorption behavior of gallium (Ga), vanadium (V), and aluminum (Al) ions on a strong acidic styrene cation exchange resin (JK resin) from a simulated Bayer solution was systematically investigated by static experiments. The results showed that the optimum conditions for separating Ga from V and Al were at low temperatures and short contact times, with 78.30%, 15.16%, and 6.63% of the adsorption efficiency at 25 °C and 60 min, respectively, for Ga, V, and Al. The adsorption kinetics of Ga^3+^ conformed to the pseudo-second order model, and the static saturation adsorption capacity was 18.25 mg/g. The Langmuir model fitted the adsorption isotherm of gallium well, and the maximum adsorption capacity was 1.11 mg/g at 25 °C. FT-IR spectroscopy and XPS showed that the mechanism of the Ga^3+^ adsorption was only related to the interaction of the oxygen atoms of the amide oxime group (C=NOH). The separation of Ga, V, and Al can be achieved by desorbing 98% of Al with low concentrations of ammonia and 90% of Ga with low concentrations of hydrochloric acid. The results indicate that JK resin is an efficient adsorbent for separating gallium and vanadium in alkaline solutions.

## 1. Introduction

Gallium is a rare metal element, known as “the backbone of the electronics industry” [1]. Gallium and its derivates are mainly used in semiconductors, catalysis, medicine, and other fields, with the semiconductor industry accounting for 80–85% of the total consumption [2,3,4]. Gallium is found in a homogeneous form in bauxite and sphalerite and is usually isolated and extracted as a by-product [5,6]. Although the annual production of gallium is increasing yearly at an average annual rate of 7.4%, demand for gallium is also increasing rapidly with the booming semiconductor industry [7,8]. By 2050, the demand for gallium will be around tenfold. Therefore, it is of great significance to find an efficient method for recovering gallium from gallium-containing solutions.

Currently, there are many methods for recovering gallium from solutions, among which are chemical precipitation, the electrolysis method, solvent extraction, and the ion exchange method [9,10]. The chemical precipitation method is also known as the calcium carbonate alumina method [11,12]. The process is characterized by a gradual carbonation process, which involves treating aluminate solutions with carbon dioxide or lime in several controlled stages to produce a co-precipitation of aluminum–gallium, followed by the dissolution of the gallium with a NaOH solution. The chemical precipitation method is simple to operate, but it requires a long time and the product is not that pure. It also generates a large amount of difficult-to-handle solid waste in the form of 3CaO·Al_2_O_3_·6H_2_O [13]. Electrolysis is a method that utilizes liquid mercury as the electrolytic cathode to electrochemically precipitate gallium [14]. The low electronegativity of hydrogen ions compared to gallium ions makes it theoretically difficult to ionize gallium ions in the solution. However, the high hydrogen overvoltage of liquid mercury allows the formation of a gallium amalgam, thereby reducing the deposition potential and enabling gallium precipitation [15]. Nevertheless, amalgam electrolysis has unavoidable disadvantages, such as a long electrolysis time, difficult solution stirring, high mercury consumption, and environmental hazards. As a result, it has been universally banned by countries worldwide. The solvent extraction method is extracting Ga from a solution into an organic solvent and can be divided into the acidic extraction system and the alkaline extraction system based on the different acidity or alkalinity of the extractant [16,17]. Zhang et al. proposed a process for extracting Fe with N235 and gallium with Cyanex272, and 99.9% of gallium could be recovered from a strong acid solution by a four-stage countercurrent extraction [18]. Liu et al. used N235 and TBP as organic systems to effectively recover 99.0% of Ga(III) from iron-removing oxalate leachate [19]. Sankum Nusen et al. selected a collaborative solvent extraction consisting of LIX 63 and Versatic 10 for the recovery of Ga(III) from a sulfate leaching solution, extracting 92% of Ga(III) with a single contact at a pH of 3.0 [20]. Helgorsky J et al. proposed the use of Kelex-100 as an extractant for the recovery of gallium from alkaline solutions [21]. Gallium was extracted from Bayer solution by Raiguel et al. using two 1,2 3-triazole ionic liquids in conjunction with Kelex^®^ 100, illustrating the advantageous impact of ionic liquids on the extraction procedure [22]. However, because of its drawbacks, including extractant loss, a difficult synthesis procedure, the high cost, and solution contamination, solvent extraction is not appropriate for industrial manufacturing.

Compared with other methods for gallium separation, ion exchange is considered the most advantageous method due to its low pollution and cost, short process, high selectivity, lack of need to add any reagents to the solution, etc. Joris Roosen et al. immobilized 8-hydroxyquinoline and 8-hydroxyquinaldine onto chitosan–silica materials for the adsorption of gallium from solutions. After seven adsorption/desorption cycles, the adsorption efficiency was still higher than 97% [23]. Magdy Khalil et al. synthesized nano-composite materials of γ-irradiated titanium dioxide and polyacrylonitrile (PAN) for the separation of gallium, strontium, and rubidium ions from aqueous solutions. The results indicated that PAN@TiO_2_ nano-composite materials have significant potential for treating actual groundwater samples [24]. Meng et al. prepared a novel porous resin using 2-ethylhexyl phosphonic acid mono-2-ethylhexyl ester (P507) and tri-n-butyl phosphate (TBP) as carriers through a vacuum impregnation–drying method. The synthesized adsorbent demonstrated an excellent gallium adsorption capacity and a high separation capability in a low concentration sulfuric acid solution [25]. Pushap Raj et al. prepared different p-quinone derivatives functionalized cellulose substrates through esterification reactions and used them for the selective extraction of gallium from dilute acidic solutions. Under pH = 3 conditions, 3,4-dihydroxybenzoic acid (P1), 3,4,5-trihydroxybenzoic acid (P2), and 3,4-dihydroxyhydrocinnamic acid (P3) exhibited maximum adsorption capacities for Ga of 31.50, 14.22, and 29.50 mg/g, respectively [26]. Li et al. synthesized UiO-66 series metal–organic skeleton composites by the solvent heat method for the recovery of gallium from a solution. The adsorption mechanism was mainly ion exchange to form chelates with an electrostatic interaction [27]. However, the above experimental materials were mostly used in acidic solutions, while the synthesis of materials is more complex and has not been industrialized. Currently, more than 90% of gallium is extracted from Bayer solution, which is a strong alkaline solution with 3–8 mol/L of NaOH. The above material is difficult to use to recover gallium from Bayer solution [28]. Therefore, it is urgent to seek a resin with alkali resistance and separation performance.

In this paper, several typical commercial resins were screened. The optimal process parameters for gallium adsorption onto JK resin were determined through single-factor experiments. Adsorption kinetics models were used to fit the process of gallium adsorption onto JK resin, thus calculating the adsorption capacity for a single adsorption of gallium. Adsorption isotherms were employed to calculate the saturation capacity of the gallium adsorbed onto the JK resin. Fourier transform infrared spectroscopy (FTIR) and X-ray photoelectron spectroscopy (XPS) reveal the functional groups of the JK resins and adsorption models combined with gallium. This paper is expected to promote the faster recovery of gallium, reducing the current gallium supply and demand tension.

## 2. Material and Methods

### 2.1. Material

The strong acidic styrene cation exchange resins (JK resins) were purchased from Zhejiang Jinshan New Material Co., Ltd. (Jiaxing, China). The Amberlite specialty resins were purchased from Solarbio Biotechnology Ltd. (Beijing, China). The D201, D301, and D701 resins; gallium oxide (Ga_2_O_3_); divanadium pentoxide (V_2_O_5_); sodium aluminate (NaAlO_2_); sodium hydroxide (NaOH); and nitric acid (HNO_3_) were acquired from Chengdu Ding Sheng Chemical Co., Ltd. (Chengdu, China). All the reagents were utilized immediately without preparation.

### 2.2. Characterization of the JK Resin

Scanning electron microscopy was used to determine the interior surface morphology of the resin (SEM, Hitachi Regulus 8230, Tokyo, Japan). The resin functional groups were identified using a Fourier transform infrared spectrometer with a KBr model in the range of 4000–400 cm^−1^ (FT-IR, Spectrum Two Li10014, Waltham, MA, USA). X-ray photoelectron spectroscopy (XPS, Thermo Scientific K-Alpha, Waltham, MA, USA) was used to examine the elements, including their chemical states, on the resin’s surface. X-ray photoelectron spectroscopy with monochromatic X-ray Al Kα radiation (1486.6 eV) (XPS, Thermo Scientific K-Alpha, Waltham, MA, USA) was employed for the identification of the elements, including their chemical states, present on the surface of a resin.

### 2.3. Adsorption and Desorption Experiment

The solution composition used in the experiment is shown in Table 1. The adsorption and desorption properties of Ga(III) in the simulated Bayer solution by JK resin were investigated. An electronic balance was used to weigh 3 g of JK resin, which was then put into a 100 mL beaker. In total, 20 mL of the simulated Bayer solution was accurately absorbed, and the beaker was placed in a water bath (with no special circumstances). It was stirred for 60 min at a fixed speed, and then the solid and liquid solutions were separated to obtain the adsorbed solution and the resin loaded with metal ions. The resin loaded with ions was placed in a 100 mL beaker. In total, 20 mL of the desorption solution was accurately absorbed. The beaker was placed in the water bath and stirred at a fixed speed for 30 min, followed by the separation of the solid and liquid (with no special circumstances). ICP-OES was employed to detect the concentration of the metal ions in the solution before adsorption, after adsorption, and after desorption. The calculation formulas of the adsorption capacity (q), adsorption rate (R), and desorption rate (DE) are shown as follows [4,29].

(1) $$q=\frac{{(C}_{o}-C_{t})\times V}{m}$$

(2) $$R=\frac{C_{o}-C_{t}}{C_{o}}\times 100\%$$

(3) $$DE=\frac{C_{1}V_{1}}{q\times m}\times 100\%$$

where C_o_, C_t_, and C_1_ are the initial concentrations of Ga(III), the concentrations of Ga(III) ions at specific times, and the concentrations of Ga(III) in the desorption solution (mg/L). V and V_1_ represent the volumes of the adsorption and desorption solutions (mL), m represents the mass of the JK resin (g), and q is the adsorption capacity at a specific time (mg/g).

### 2.4. Adsorption Isotherm Model

The adsorption isotherm is capable of characterizing the distribution of Ga(III) between the solution and the JK resins under various Ga(III) concentrations in the solution. Commonly utilized adsorption models are the Langmuir model and the Freundlich model. The Langmuir model presumes that the adsorption is chemical, while the Freundlich model assumes that the adsorption is physical.

The Langmuir isotherm adsorption model is as follows:

(4) $$\frac{C_{e}}{q_{e}}=\frac{C}{q_{m}}+\frac{1}{K_{L}q_{m}}$$

where q_e_ is the adsorption capacity of Ga(III) in the amidoxime material at the adsorption equilibrium (mg/g); C_e_ is the concentration of Ga(III) in the solution at equilibrium (mg/L); q_m_ is the saturated adsorption capacity (mg/g); and K_L_ is the Langmuir model equilibrium constant (L/mg).

The Freundlich isotherm adsorption model is as follows:

(5) $${lnq}_{e}={lnK}_{f}+\frac{{lnC}_{e}}{n}$$

where q_e_ is the adsorption capacity of Ga(III) in the resin at the adsorption equilibrium (mg/g); C_e_ is the concentration of Ga(III) ions in the solution at equilibrium (mg/L); K_f_ is the Freundlich equilibrium constant (mg/g); and n is the Freundlich equilibrium constant.

### 2.5. Adsorption Kinetics Model

The large-scale application of adsorption materials in industrial production hinges on their superior kinetic performance. To investigate the impact of reaction time on the adsorption of Ga(III) by JK resins, kinetic equations (pseudo-first order kinetics and pseudo-second order kinetics) were introduced to conduct the fitting and analysis on the adsorption rate of Ga(III) by JK resins and ascertain the mechanism of the adsorption process. The expressions of the kinetic equations are as follows.

The pseudo-first order kinetic equation is as follows:

(6) $${ln(q}_{e}-q_{t})={lnq}_{e}-k_{1}t\;$$

where q_e_ is the adsorption capacity of the metal ions in the adsorbed material at the adsorption equilibrium (mg/g); q_t_ is the adsorption capacity of the metal ions in the adsorbed material at time t (mg/g); k_1_ is a pseudo-first order kinetic reaction rate constant (min^−1^); and t is the reaction time (min).

The pseudo-second order kinetic equation is as follows:

(7) $$\frac{t}{q_{t}}=\frac{1}{k_{2}q_{e}^{2}}+\frac{t}{q_{e}}$$

where q_e_ is the adsorption capacity of the metal ions in the adsorbed material at the adsorption equilibrium (mg/g); q_t_ is the adsorption capacity of the metal ions in the adsorbed material at time t (mg/g); k_2_ is a pseudo-second order kinetic reaction rate constant (mg/(g·min)); and t is the reaction time (min).

## 3. Results and Discussion

### 3.1. Screening of Adsorbents

**Figure 1:**
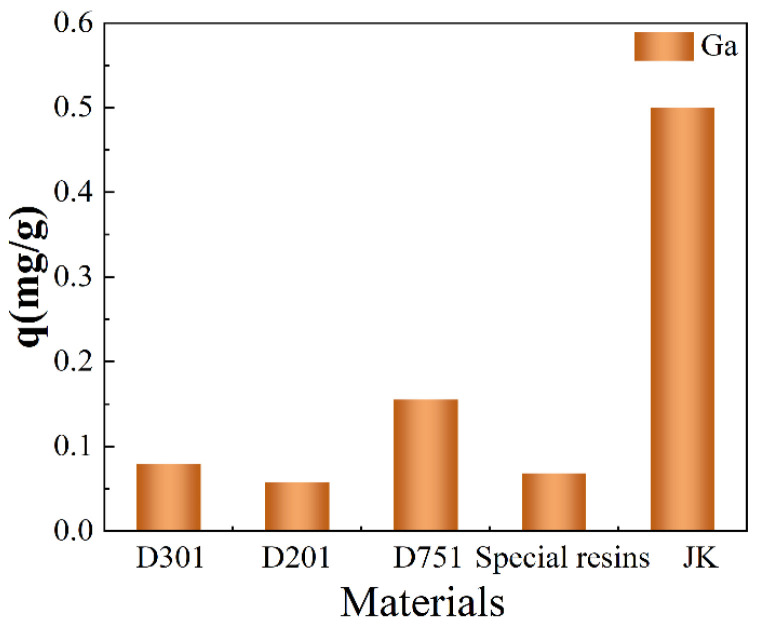
Effect of different resins on gallium adsorption.

Different kinds of resins, including D201, D301, D751, Amberlite specialty resins, and JK resins, were applied to adsorb the gallium from the simulated Bayer solution. It can be seen from Figure 1 that, in the simulated Bayer solution, D301, D201, D751, and the Amberlite special resin had poor adsorption efficiencies on gallium with an adsorption capacity below 0.15 mg/g. In contrast, the JK resins showed an excellent adsorption performance on gallium, with an adsorption capacity above 0.50 mg/g. Therefore, the JK resin was selected for further adsorption experiments on gallium.

### 3.2. Adsorption Experiments

**Figure 2:**
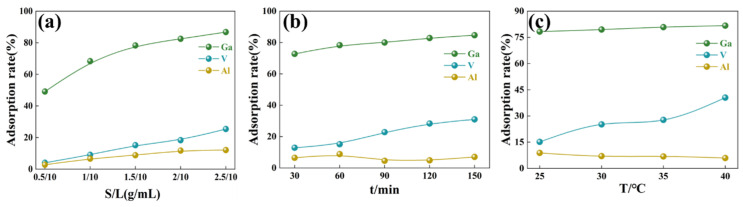
The effect of the process parameters on the adsorption of gallium, vanadium, and aluminum by JK resin: (**a**) solid–liquid ratio, (**b**) time, and (**c**) temperature.

Figure 2 shows the effect of the process parameters on the adsorption efficiencies of Ga(III), V(V), and Al(III) by JK resin. The increased mass of the JK resin facilitates the adsorption of gallium in Figure 2a. The adsorption efficiencies of the JK resin for gallium, vanadium, and aluminum were 49.12%, 4.06%, and 2.80%, respectively, with a solid–liquid ratio of 0.5/10 g/mL. The adsorption efficiencies of the JK resin for gallium, vanadium, and aluminum increased to 86.77%, 25.37%, and 12.06%, respectively, with the solid–liquid ratio increasing to 2.5/10 g/mL. Considering the subsequent separation problem, the solid–liquid ratio was selected as 1.5/10 g/mL, at which the adsorption efficiencies for Ga(III), V(V), and Al(III) were 78.30%, 15.16%, and 6.63%, respectively. The effect of time on the Ga(III) adsorption by the JK resin is shown in Figure 2b. The adsorption efficiency of gallium was 72.73% within 30 min, indicating that the JK resin had a good adsorption kinetic performance on gallium. The increase in adsorption time favored the adsorption of V(V), with a change in adsorption efficiency from 12.91% to 31.01%. Therefore, 60 min was chosen as the optimal adsorption time for the Ga adsorption by the JK resin. The temperature had a negligible effect on the adsorption of Ga by the JK resin, with the gallium adsorption increasing from 78.30% to 81.68% in the temperature range of 25 to 45 °C in Figure 2c. However, the adsorption efficiency of vanadium increased from 15.16% to 40.51%. The higher temperature was more favorable for the adsorption of V(V) by the JK resin, while the adsorption efficiency of Al(III) was almost unchanged. Therefore, 25 °C was chosen as the adsorption temperature for the Ga adsorption by commercial JK resins.

### 3.3. Adsorption Isotherm

**Figure 3:**
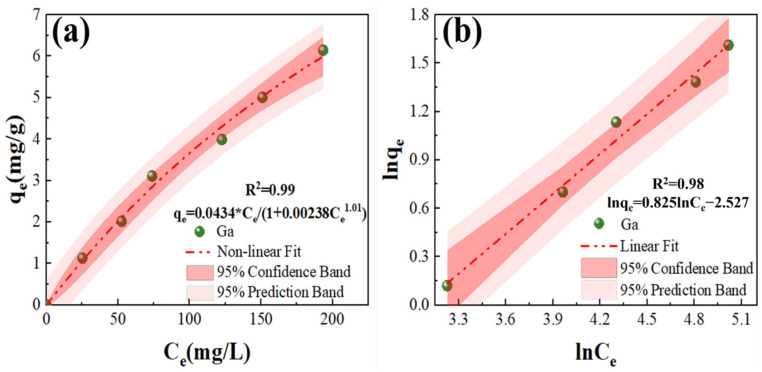
Adsorption isotherms’ fitting of Ga(III) with JK resins: (**a**) Langmuir, and (**b**) Freundlich.

The Langmuir and Freundlich models are frequently employed to fit adsorption isotherms [30,31]. Figure 3 depicts the isotherm of gallium on JK resin. The JK resin’s adsorption capability grew with its original concentration. The Langmuir model had a fitting coefficient of 0.99, which was closer to 1 than the Freundlich model. The saturated adsorption capacity estimated from the Langmuir model was 18.25 mg/g, but the Freundlich model yielded 0.08 mg/g, which diverged from the experimental value. As a result, the Langmuir model was better suited to describing the experimental findings than the Freundlich model. Table 2 shows a comparison of the adsorption capabilities of JK resin with other adsorbents. The saturation adsorption capacities of the commercial resins LSC600 and LSC700 were 3.6 mg/g and 12.3 mg/g [32,33], respectively, both lower than the JK resin, indicating the future potential of JK resin in industrial applications.

### 3.4. Adsorption Kinetics

**Figure 4:**
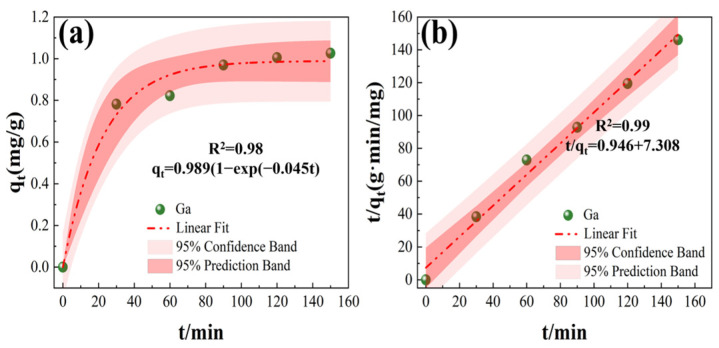
Adsorption kinetic fitting of Ga(III) with JK resins: (**a**) pseudo-first order model, and (**b**) pseudo-second order model.

The pseudo-first order and pseudo-second order kinetic models are often used to describe the adsorption process of ionic resins, and the fitting results of kinetics are shown in Figure 4 and Table 3. As seen in Figure 4a, the pseudo-first order kinetic model fit is not very good, with a fitted parameter R^2^ of 0.98 and a calculated q_o_ value of 0.97 mg/g. The fitted theoretical data do not match the actual adsorption situation, indicating that the adsorption process of Ga(III) ions in simulated Bayer solutions by JK resin does not follow the pseudo-first order kinetic model. Figure 4b shows that the fitting results of the pseudo-second order kinetic model are better than those of the pseudo-first order kinetic model. The fitting parameter R^2^ is 0.99, closer to 1 than that of the pseudo-first order kinetic model, and the calculated q_o_ value is 1.11 mg/g. The pseudo-second order kinetic model closely approximates the actual data (1.09 mg/g). Therefore, the JK resin adsorption process of Ga(III) ions from the Bayer solution is mainly chemical adsorption, in which the effect of physical adsorption can be ignored. The gallium extraction resin currently used in industry is LSC600 in China, and the kinetic data of the JK resin and LSC600 resin are listed in Table 3. The results show that the fitting data of the two kinetic models for the adsorption of gallium by the JK resin are higher than those of the LSC600 resin, indicating that the JK resin should be expected to replace the LSC600 resin to more efficiently recover gallium from Bayer solution.

### 3.5. Desorption Study

**Figure 5:**
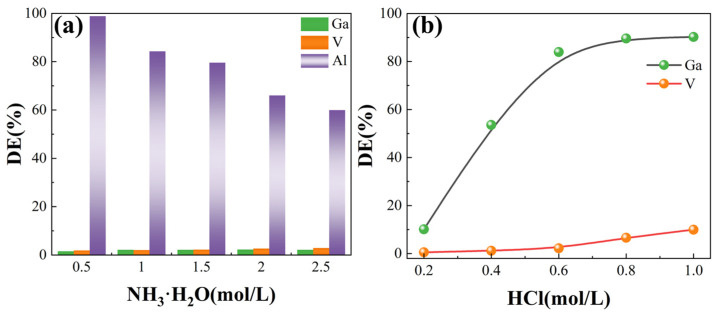
The desorption study of JK resin: (**a**) ammonia, and (**b**) hydrochloric acid.

The above results show that Al(III) and V(V) are also adsorbed by the JK resin. The ammonia solution was used to elute the Al(III) ions adsorbed by the JK-Ga resin, and the effect of different ammonia concentrations on Al(III) was investigated, as shown in Figure 5a. More than 95% of the Al(III) could be desorbed by 0.5 mol/L of ammonia. In contrast, a higher concentration of ammonia was less effective in the desorption of Al((III). The ammonia concentration had little effect on Ga(III) and V(V), with fewer than 10% of loss. This research shows that an acidic solution can desorb the adsorbed ions effectively. The Ga(III) on the JK-Ga resin was desorbed by an acid solution, and the influence of the hydrochloric acid concentration on the desorption of Ga(III) was studied, as shown in Figure 5b. The concentration of the hydrochloric acid solution is positively correlated with the desorption efficiency of the Ga(III). When the concentration of hydrochloric acid was 0.2 mol/L, the desorption efficiency of the Ga(III) ion was only 10.11%. When the concentration of hydrochloric acid increased to 1 mol/L, the desorption efficiency of Ga(III) reached 90.20%, while the desorption efficiency of the V(V) ion was only 9.96%. The effective separation of Ga(III) and V(V) can be achieved. The principle of the desorption of Ga(III) from a JK-Ga resin in an acid solution is that the acidic environment can make the amidoxime group effectively replace the Ga(III) at the binding site.

### 3.6. Resin Recyclability

**Figure 6:**
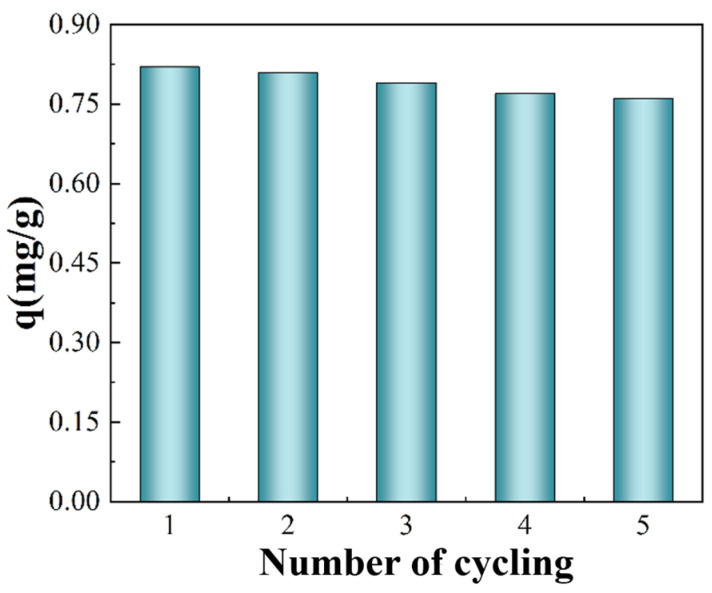
Cycling properties of JK resin.

The excellent recyclability of resins is one of the indicators of the application of resins in the industry. The recyclability performance of JK resin for the absorption of gallium was investigated through the above adsorption–twice desorption cycle experiment, and the results are shown in Figure 6. After five adsorption–desorption operations, the JK resin had an adsorption capability of 0.76 mg/g gallium. The JK resin’s initial adsorption capacity was 0.82 mg/g, and, after five cycles, it fell by 0.06 mg/g, showing that commercial JK resin has a good recycling ability.

### 3.7. Resin Characterization

**Figure 7:**
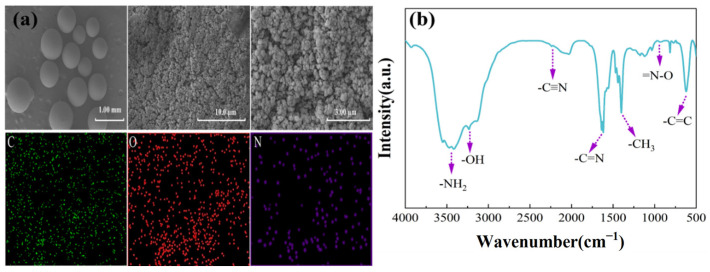
Characterization of JK resins: (**a**) SEM, and (**b**) FT-IR.

The JK resin was characterized to investigate the microscopic morphology and functional groups of the resin, and the results are shown in Figure 7. The resin comprised opaque spherical particles with varying sizes. The elements mapping indicated that the resin was composed of C, N, and O. With increasing magnification, a rougher surface could be observed. Under high-magnification electron microscopy, the resin was observed to be a loose and porous structure in Figure 7a. Figure 7b shows an absorption peak of -NH_2_ at 3440 cm^−1^, C≡N at 2234 cm^−1^, N-O at 934 cm^−1^, and C=N at 1650 cm^−1^; all the signs showed that JK resin had amidoxime functional groups (C=NOH and -NH_2_) [34,35].

### 3.8. Adsorption Mechanism

**Figure 8:**
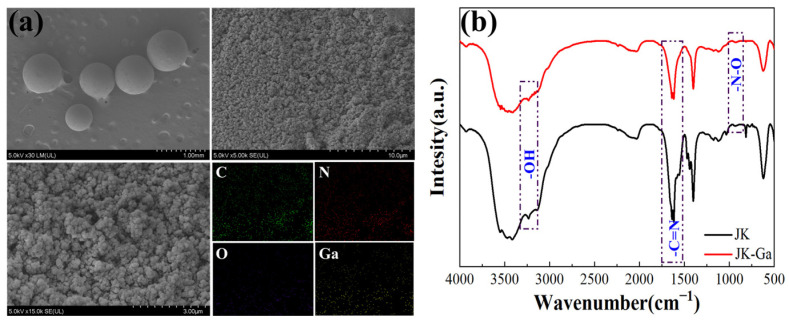
Characterization of JK-Ga resins: (**a**) SEM, and (**b**) FT-IR.

Figure 8 shows the scanning electron microscopy and infrared spectra of the Ga-enriched JK resin after adsorption. The resin morphology did not change after adsorption, and the elements mapping showed that the Ga ion was successfully loaded onto the resin. The infrared spectrum of the JK resin before and after the gallium ion adsorption was compared, and the results are shown in Figure 8b. They show that the N-O spectral peak shifted from 934 to 921 cm^−1^ and the broad peaks of -OH and -NH_2_ at 3440 cm^−1^ became more widely dispersed, which could be attributed to the changes in Ga(III) and C=NOH [36,37].

**Figure 9:**
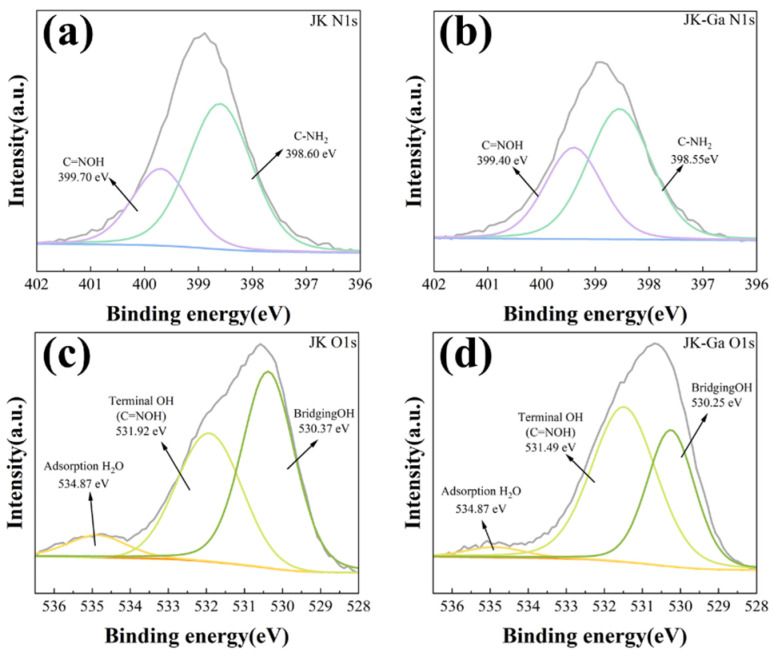
XPS’ N1s and O1s of HAOPAN resin before and after adsorption.

The XPS characterization of the JK resin before and after adsorption are shown in Figure 9. In Figure 9a, the N1s spectrum in the JK resin is separated into two spectral peaks at 398.60 eV and 399.70 eV, which correspond to the nitrogen of the NH2 species (C-NH_2_) and the nitrogen of the oxime. In Figure 9c, the O1s spectra in the JK resin can be split into three peaks—530.37 eV, 531.92 eV, and 534.87 eV—which are attributable to the bridging OH, terminal OH (C=NOH), and adsorption H_2_O, respectively [4,37]. The terminal OH (C=NOH) peak in the Ga-loaded JK resin shifted significantly, with the binding energies in the N1s and O1s spectra shifting from 399.70 eV and 531.92 eV to 399.40 eV and 531.49 eV, respectively, indicating that the oxygen-containing functional group (C=NOH) of the JK resin pools with Ga, which is consistent with the FT-IR results [37].

**Figure 10:**
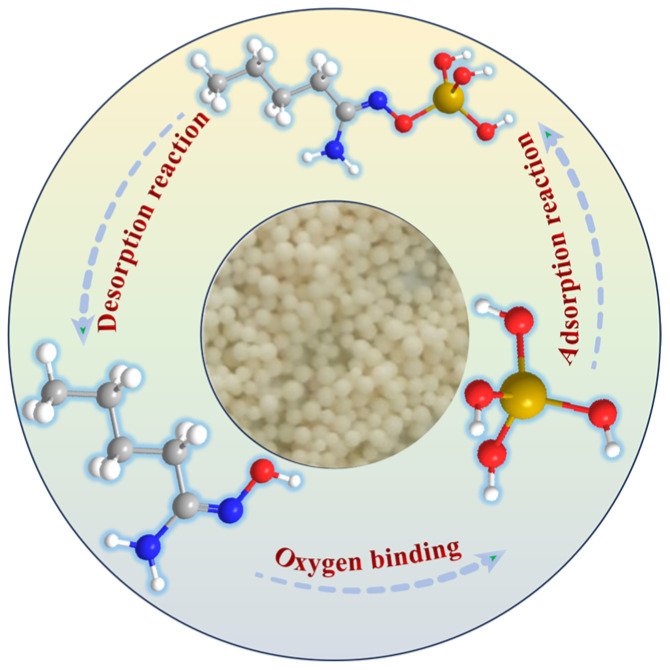
Schematic diagram of gallium adsorption by JK resin.

The form of the gallium ions in a solution is related to the pH of the solution, with a pH of less than 2 leading to a form of Ga^3+^ and a pH greater than 11 leading to a form of [Ga(OH)_4_]^−^ [38]. The alkaline concentration of the simulated Bayer solution was 3 mol/L, hence confirming that the gallium ions in the Bayer solution existed in the form of [Ga(OH)_4_]^−^. Based on the structural analysis of the JK resin, the adsorption model shown in Figure 10 could be obtained. Based on the metal ion adsorption mode of the amidoxime resin, the oxygen-bonding mode was chosen. When O is coupled to Ga, the released H^+^ combines with OH^−^ to form H_2_O from [Ga(OH)_4_]^−^. As a result, JK resin can be regarded as an excellent gallium ion adsorbent in industrial production.

## 4. Conclusions

This paper investigated the adsorption–desorption performance of JK resin for gallium separation from a simulated Bayer solution. The effects of the liquid–solid ratio, adsorption time, and temperature on the adsorption separation were investigated, and the experimental data were fitted by adsorption kinetics, adsorption thermodynamics, and adsorption isotherms. After adsorption, the desorption of the resin was investigated, using ammonia–hydrochloric acid as the eluent to explore the effect of the eluent concentration on the desorption effect. Under the optimized conditions, the gallium, vanadium, and aluminum adsorptions were 78.30%, 15.16%, and 6.63%, respectively. The kinetics of the gallium adsorption by the JK resin were consistent with the pseudo-second order model, with a static saturation adsorption of 18.25 mg/g showing that the process was primarily chemisorption. The Langmuir model fitted the gallium adsorption isotherm well, with a maximum adsorption of 1.11 mg/g of gallium at 25 °C. FT-IR and XPS showed that the adsorption mechanism of gallium was only related to the interaction of oxygen atoms of the amidoxime group (C=NOH). Two-step desorption can achieve the separation of gallium, vanadium, and aluminum. JK resins have excellent cycling properties and are expected to replace LSC600 resins in industrial production.

## Figures and Tables

**Table 1:** Simulating the composition of the Bayer solution.

Solution Components	Ga (mg/L)	V (mg/L)	Al (g/L)	NaOH (mol/L)
Concentration	200	120	30	3

**Table 2:** Comparison of Ga(III) sorption capacities (mg g^−1^) for different materials.

Adsorbents	C_o_ (mg/L)	pH	q (mg/g)	Ref
LSC600 resin	25	13.7	3.6	[33]
LSC700 resin	200	14	12.3	[32]
JK resin	200	14.48	18.25	This work

**Table 3:** Kinetic parameters of JK resin and LSC600 resin.

Materials	Pseudo-First Order Model (mg/g)	k_1_ (min^−1^)	Pseudo-Second Order Model (mg/g)	k_2_ (mg/(g·min))	Ref
LSC600 resin	0.39	0.0176	0.43	0.0507	[33]
JK resin	0.989	0.045	1.11	0.1212	This work

## Data Availability

The original contributions presented in the study are included in the article, further inquiries can be directed to the corresponding author.
